# Case report: Tongue metastasis as an initial sign of clear cell renal cell carcinoma and its prognosis

**DOI:** 10.3389/fonc.2024.1473211

**Published:** 2025-01-27

**Authors:** Shuo Liu, Hongyun Liu, Bowen Weng, Sichuan Hou

**Affiliations:** ^1^ School of Clinical Medicine, Shandong Second Medical University, Weifang, China; ^2^ Department of Pathology, Qingdao Municipal Hospital, Qingdao, Shandong, China; ^3^ Department of Urology, Qingdao Municipal Hospital, Qingdao, Shandong, China

**Keywords:** clear cell renal cell carcinoma, metastasis, tongue, cytoreductive nephrectomy, metastasectomy, targeted therapy, immunotherapy

## Abstract

Clear cell renal cell carcinoma (ccRCC) is the most prevalent and lethal subtype of renal cell carcinoma (RCC), characterized by a poor prognosis and a high likelihood of distant metastasis. Nonetheless, metastasis of ccRCC to the tongue remains rare. Diagnosing and planning treatment for patients who initially present with tongue metastasis can be particularly challenging, as few cases have been reported in the literature. We present a case of a 62-year-old man who presented with a painful lump on the right anterior border of his tongue. Histological examination revealed lobulated and nested epithelial cell clusters with moderate dysplasia and frequent mitotic figures within the lamina propria. Immunohistochemistry showed positivity for vimentin, CD10, PAX-8, and epithelial membrane antigen (EMA), but negativity for PAX-2, calponin, S-100 protein, periodic acid-Schiff with diastase (PAS-D), P63, P40, and CK7, confirming the diagnosis of ccRCC metastasis to the tongue. After comprehensive evaluation and multidisciplinary team consultation, the patient underwent cytoreductive nephrectomy (CN), metastasectomy, and targeted therapy. According to the Response Evaluation Criteria in Solid Tumors (RECIST) Version 1.1, the patient maintained stable disease (SD) during systemic treatment. Unfortunately, treatment was discontinued due to adverse drug reactions, and the patient was transitioned to palliative care. His disease progressed to progressive disease (PD), and he ultimately succumbed to systemic infection, with a progression-free survival (PFS) of approximately 15 months. This case highlights the urgent need for improved therapeutic strategies to manage symptoms and prolong survival in patients with this rare metastatic presentation.

## 1 Introduction

Renal cell carcinoma (RCC) is the third most prevalent urological malignancy, accounting for approximately 2.2% of all cancers worldwide (1). Clear cell renal cell carcinoma (ccRCC) is the most common and aggressive form of RCC, characterized by a poor prognosis and a high propensity for distant metastasis. Approximately 45% of patients diagnosed with RCC either have or are at risk of developing distant metastasis, with common sites including the lungs (45.2%), bones (29.5%), lymph nodes (21.8%), liver (20.3%), adrenal glands (8.9%), and brain (8.1%) (2, 3). Metastasis to the oral cavity is rare, accounting for around 1% of oral malignancies (4).

While tongue metastasis following kidney cancer surgery has been documented (5), there are few instances where lingual metastasis is the initial presentation of ccRCC before diagnosis and subsequent systemic treatment. This report describes a case of metastatic ccRCC that originally presented as a painful lump on the front right edge of an elderly man’s tongue and provides a comprehensive discussion on the diagnosis and management of this condition and follow-ups.

## 2 Case description

**Figure 1:**
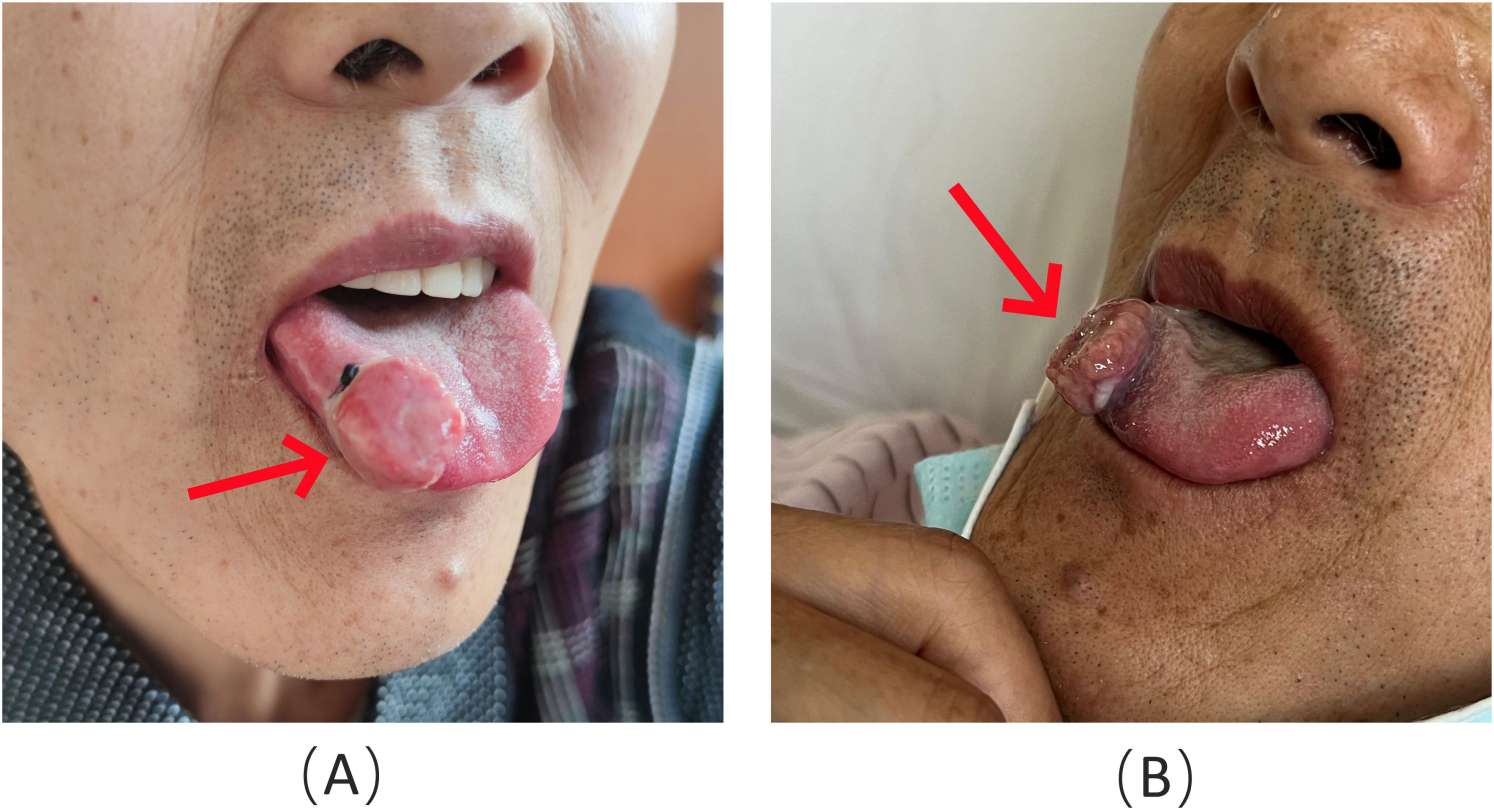
A protruding bright red mass (red arrow) on the right front border of the tongue.

**Figure 2:**
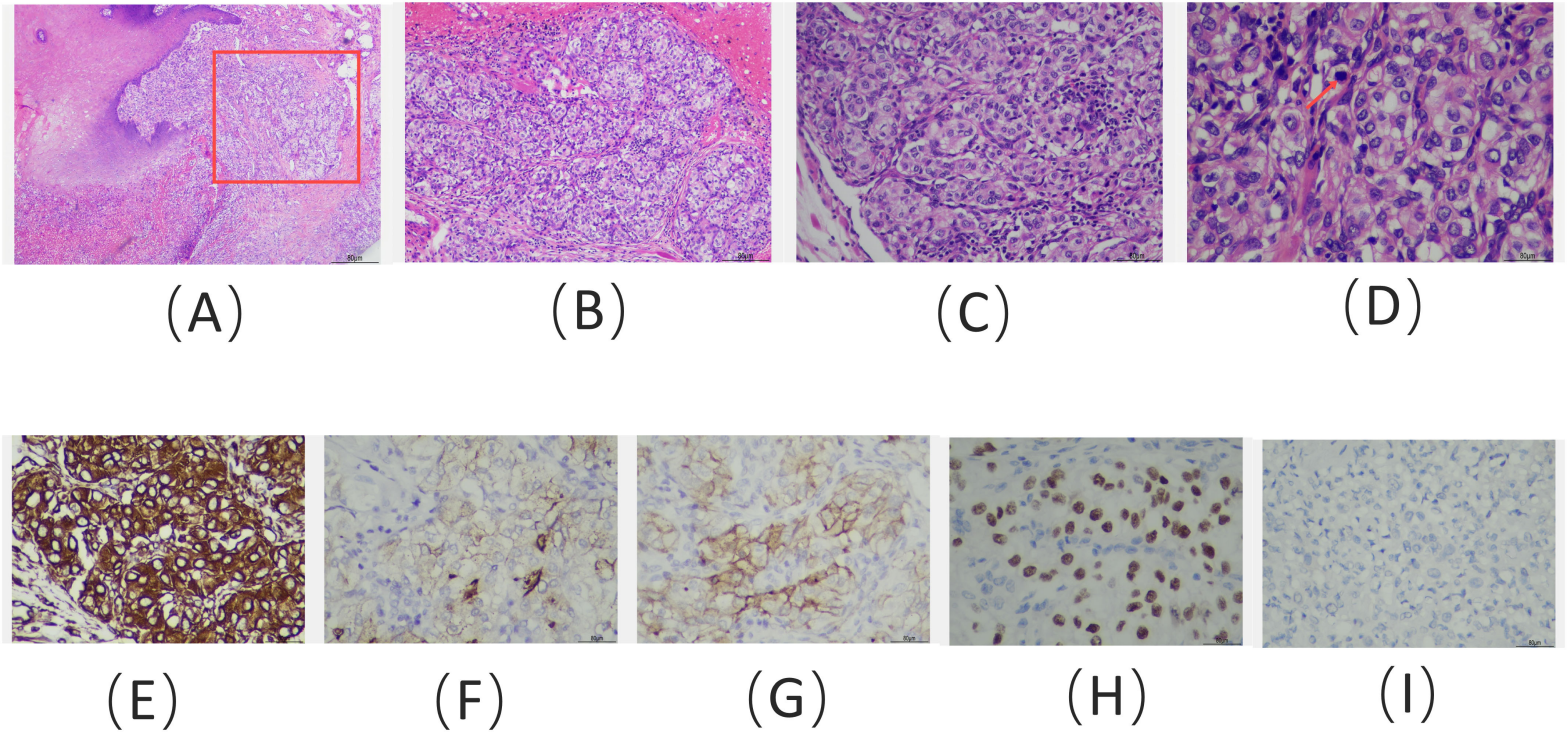
Hematoxylin and eosin staining of the tongue lesion reveals: **(A)** clusters of tumor cells located within the mucous membrane (red box) and submucosal layer, adjacent to the squamous epithelial tissue of the tongue (× 100); **(B)** tumor cells organized in clusters, supported by a dense capillary network (× 100); **(C)** tumor cells with large, hyperchromatic nucleoli (× 200); and **(D)** tumor cells exhibiting clear cytoplasm and pathological mitotic figures (red arrow) (× 400). Immunohistochemical staining results: positive staining for **(E)** vimentin, **(F)** EMA, **(G)** CD10, and **(H)** PAX-8, with negative staining for **(I)** PAX-2 (immunohistochemistry; **E–I**, × 400).

**Figure 3:**
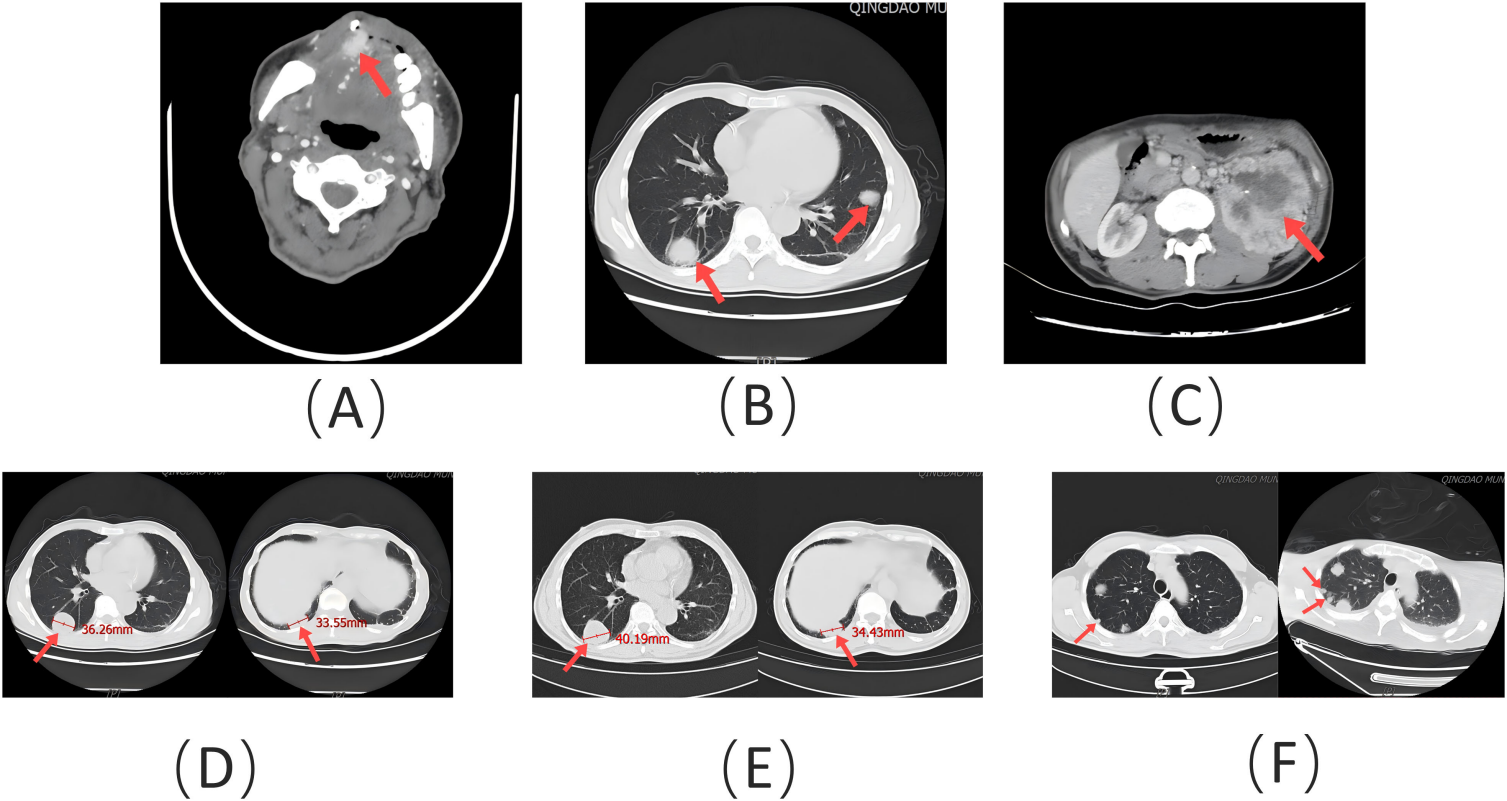
**(A)** Contrast-enhanced head and neck CT scan showing an unevenly enhanced lesion (red arrow) on the patient’s tongue. **(B)** Chest CT scan demonstrating spiculated nodules (red arrow) of varying sizes in both lungs. **(C)** Abdominal contrast-enhanced CT scan illustrating an irregular mass (red arrow) in the left kidney. **(D)** Size of target lesions (red arrow) in the lungs prior to treatment. **(E)** Size of target lesions (red arrow) in the lungs after 7 months of treatment, evaluated as stable disease (SD). **(F)** Following discontinuation of treatment, a new metastatic lesion (red arrow) appeared, and target lesions were evaluated as progressive disease (PD).

A 62-year-old man presented to the Stomatology Department of Qingdao Municipal Hospital with a painful lump on the right front border of his tongue, which had been bothering him for the past 20 days. The oral mass, initially described as painful and ulcerative, grew quickly in size over the 20-day period. The patient did not report any difficulty swallowing, changes in taste, or bleeding from the lesion. He had experienced a weight loss of approximately 3 kg over the past year. The patient had no history of smoking or alcohol use, and his medical history was unremarkable. Examination revealed a 2-cm bulging mass with a bright red color on the front right of the tongue (**Figure 1**). An excisional tissue biopsy was performed, revealing lobulated and nested epithelial cell clusters with moderate dysplasia and frequent mitotic figures in the lamina propria (**Figures 2A-D**). The lesion was initially suspected to be a malignant tumor, possibly originating from the salivary gland. A head and neck CT scan with contrast revealed an unevenly enhanced lump on his tongue (**Figure 3A**), while a chest CT identified multiple high-density nodules in both lungs, suspected to be lung metastases (**Figure 3B**). Immunohistochemical analysis showed positivity for vimentin, epithelial membrane antigen (EMA), CD10, and PAX-8, while being negative for PAX-2 (**Figures 2E-I**), calponin, S-100 protein, periodic acid-Schiff with diastase (PAS-D), P63, P40, and CK7. Based on these findings, the diagnosis was metastatic ccRCC of the tongue.

**Figure 4:**
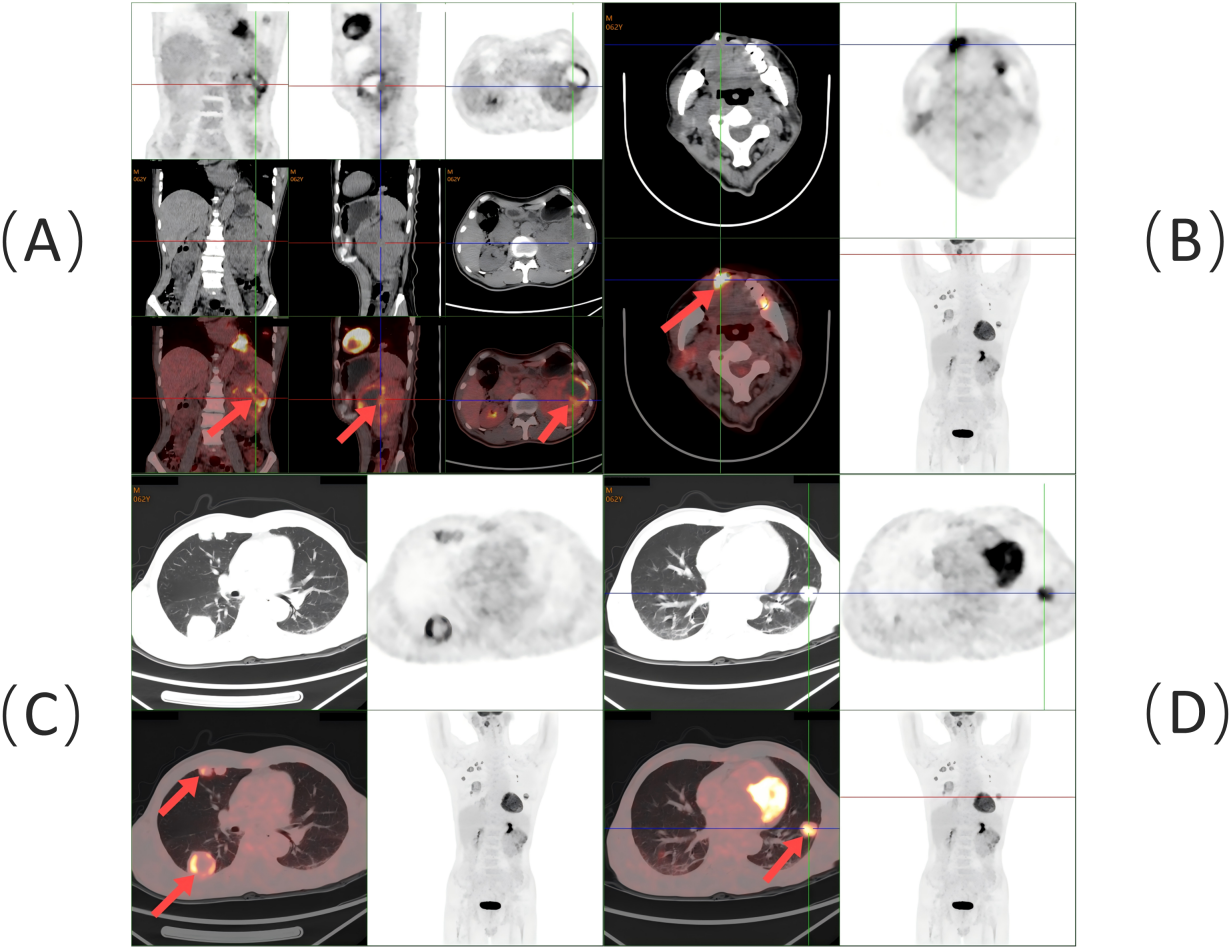
Whole-body PET/CT scan findings: **(A)** Soft tissue mass with ^18^F-FDG hypermetabolism in the left kidney (red arrow). **(B)** Soft tissue density lesion with ^18^F-FDG hypermetabolism at the right anterior border of the tongue (red arrow). **(C)** Multiple ^18^F-FDG hypermetabolic nodules and masses in the right lung (red arrow). **(D)** ^18^F-FDG hypermetabolic nodule in the left lung (red arrow).

Following the diagnosis, the patient underwent imaging within 2 weeks. A contrast-enhanced CT of the abdomen revealed a lumpy mass measuring approximately 9.8 cm × 8.6 cm in the left kidney, with heterogeneous enhancement typical of RCC (**Figure 3C**). A whole-body PET-CT scan confirmed ^18^F-FDG uptake in the left kidney mass, with a maximum standardized uptake value (SUVmax) of 19.2, indicating malignancy. The scan also revealed tongue metastasis (SUVmax 9.4) and bilateral lung metastases (SUVmax 4.8 and 6.5) (**Figure 4**). No other distant metastases were found, and the patient was diagnosed with metastatic ccRCC. The corrected calcium level was 2.83 mmol/L, above the normal range of 2.11–2.52 mmol/L. Hemoglobin was 171 g/L, within the normal range of 130–175 g/L. The neutrophil count was 3.43 × 10^9^/L, within the normal range of 1.8–6.3 × 10^9^/L. The platelet count was 332 × 10^9^/L, also within the normal range of 125–350 × 10^9^/L. The interval between diagnosis and treatment was less than 1 year, and the Karnofsky performance status was 90%. According to the International Metastatic RCC Database Consortium (IMDC) risk scores, the patient was classified as intermediate risk (with two risk factors) (6). Following multidisciplinary consultation, he was transferred to the Urological Surgical Department for further treatment. The patient underwent a robot-assisted cytoreductive nephrectomy (CN) there and a tongue tumor resection procedure at the Department of Oral Surgery 12 days later.

One month following CN, the patient initiated a 2-week treatment regimen of sunitinib at a dosage of 50 mg/day, followed by a 1-week interval. Additionally, the patient received toripalimab (240 mg intravenously once every 3 weeks), a humanized antiprogrammed cell death protein 1 (PD-1) IgG4k antibody. After 3 months of comprehensive treatment, the patient’s white blood cell count fell. Subsequent treatment with leukocyte-boosting medications restored the white blood cell count to normal levels. However, the count decreased again, leading to continued administration of the drug to stimulate leukocyte production. Unfortunately, the therapeutic effect was not satisfactory.

Seven months after CN, a chest CT scan revealed an increase in the size of some lesions in both lungs compared to the previous scan. The decrease in white blood cell count persisted, along with a drop in neutrophil count. Consequently, systemic treatment (ST) was discontinued, and medications were administered to increase white blood cell and neutrophil counts. After 1 month, the patient’s blood count improved, and he resumed further ST. However, ST was discontinued 2 months later due to the subsequent onset of epilepsy and intracerebral hemorrhage, and conservative treatment was administered. In the end, the patient was transferred to palliative care. Unfortunately, his condition worsened, and he passed away due to a systemic infection after approximately 3 months later. The progression-free survival (PFS) was approximately 15 months.

## 3 Discussion

Oral and maxillofacial metastases are rare for ccRCC and typically indicate a poor prognosis and low overall survival (OS) rate. While previous reports have mentioned these occurrences, few have provided detailed treatment plans and follow-up outcomes (7). This case is notable for presenting tongue metastasis as the initial sign of ccRCC and for outlining a comprehensive treatment approach with follow-up results.

The majority of tongue metastases from RCC typically present as rapidly growing masses accompanied by pain, bleeding, and signs of aggressive malignancy, such as dysphagia and dysarthria (8). In some cases, benign manifestations, such as painless submucosal nodules, may also be observed (9). It is hypothesized that tongue metastasis may be linked to hematological dissemination through Batson’s paravertebral valveless venous plexus (8). Oral metastases are typically discovered 1–7 years after the primary tumor diagnosis, although there are instances where metastatic tumors are identified before the primary tumor. PET-CT detects tumors by identifying metabolic changes, especially in the early stages when tumor cells exhibit increased glucose metabolism. This can be visualized with ^18^F-FDG, even in small tumors not detectable by conventional imaging. Metastatic ccRCC often affects areas of the mouth containing salivary glands (9). However, the morphological overlap between metastatic ccRCC and primary salivary gland tumors—such as epithelial–myoepithelial carcinoma, myoepithelial carcinoma, mucoepidermoid carcinoma, and adenoid cell carcinoma—can pose diagnostic challenges (10). Immunohistochemical staining is often crucial in these cases. The ccRCC exhibits a structure similar to that of the kidney, with nests and tubules of cells containing clear cytoplasm and a prominent network of stromal capillaries surrounding tumor clusters. Nuclear characteristics vary with tumor grade, ranging from small, round nuclei with uniform chromatin to large, ovoid nuclei with varying degrees of nucleolar prominence (11).

Tumors with myoepithelial components typically show positive immunohistochemical staining for calponin and P63, whereas metastatic ccRCC is negative. In contrast, adenoid cell carcinoma is positive for PAS-D, unlike metastatic ccRCC. Mucin staining can also help distinguish metastatic ccRCC from mucoepidermoid carcinoma (10). In metastatic ccRCC, commonly expressed immunohistochemical markers include CD10, PAX-2, PAX-8, human kidney injury molecule-1 (hKIM-1), renal cell carcinoma monoclonal antibody (RCCma), von Hippel–Lindau (VHL) tumor suppressor gene products, EMA, E-cadherin, and S-100 protein (9, 10). While some of these markers can be found in salivary gland tumors, PAX-2, PAX-8, and hKIM-1 typically show negative reactivity in such tumors (10). In this case, both PAX-2 and S-100 protein were negative. Metastatic ccRCC typically shows strong positivity for CD10 and is either negative or only locally positive for CK7, distinguishing it from clear cell papillary cystadenoma (12). Additionally, CK7 is a sensitive marker for papillary RCC (13).

Treatment options typically include CN, local therapy for RCC metastases, targeted therapy, and immunotherapy (14). Surgery aims to alleviate symptoms and discomfort in patients. In this case, the patient underwent robot-assisted CN and tongue tumor resection, followed by a combination of targeted and immunological therapies. CN involves removing the primary lesion. In the age of targeted therapy, retrospective analyses have shown that patients who undergo CN followed by targeted therapy have better survival outcomes than those who receive targeted therapy alone (15). While early CN standardization may not be recommended for individuals with poor physical condition or unfavorable prognostic indicators, it still plays a crucial role in managing metastatic RCC in patients with limited metastatic burden, those requiring palliative care, or those showing a positive response to initial systemic treatment (16). A good performance status and intermediate IMDC risk classification were predictive of OS benefit with CN (16). In this case, the patient presented with a good overall condition, an intermediate-risk IMDC classification, and the potential for complete excision of the primary tumor. This warranted immediate CN followed by ST, in line with the principles of individualized therapy. The primary tumor was successfully excised with the aid of Da Vinci surgical robots.

Therapeutic approaches for tongue metastases encompass local resection, which may be supplemented with chemotherapy and/or radiation therapy for comprehensive care (17). Surgical excision of tongue metastasis was considered a suitable local treatment for this condition. Consistent findings suggested that performing margin-free metastasectomy in metastatic RCC is associated with improved OS, cancer-specific survival (CSS), and delayed initiation of systemic treatment (14). The surgical margins in this case showed no evidence of tumor cells.

Enhanced understanding of pathogenic and disease mechanisms has led to the development of various novel drugs, including targeted therapies such as vascular endothelial growth factor (VEGF) receptor inhibitors and immunotherapy drugs like immune checkpoint inhibitors (ICIs). Sunitinib, an oral tyrosine kinase inhibitor, targets vascular endothelial growth factor receptor (VEGFR) and platelet-derived growth factor receptor (PDGFR) and is commonly used as primary therapy for late-stage RCC (18). Toripalimab is a humanized antiprogrammed cell PD-1 IgG4k antibody that has been approved in China for the treatment of kidney carcinoma. The utilization of ICIs has shown significant improvements in overall response rate (ORR) and PFS for individuals with advanced RCC, whether used in combination with VEGF inhibitors or as dual immunotherapy. Furthermore, combining ICIs with VEGF receptor inhibitors has demonstrated notable improvements in ORR, PFS, and OS for patients with metastatic ccRCC (19).

The patient presented to the Stomatology Department with a tongue lesion and was diagnosed with metastatic ccRCC involving the tongue, along with bilateral lung metastases. The IMDC risk was classified as intermediate. Despite undergoing surgical intervention and treatment with sunitinib plus toripalimab, the patient’s pulmonary metastases continued to progress after 7 months of CN. However, the total diameter of the target lesions did not increase by at least 20%. According to the Response Evaluation Criteria in Solid Tumors (RECIST) Version 1.1, the evaluation of target lesions was classified as stable disease (SD) (**Figures 3D, E**) (20). When ST was discontinued, the evaluation of target lesions was classified as progressive disease (PD) due to the appearance of a new target lesion in the right lung (**Figure 3F**). Therefore, ST proved to be meaningful. The endpoint was PFS, which was approximately 15 months. The primary negative responses observed during the course of treatment included a reduction in white blood cell count and a decrease in neutrophil count, epilepsy, and intracerebral hemorrhage. The patient ultimately passed away due to a systemic infection.

Instances of primary manifestations, such as tongue metastasis in metastatic ccRCC, are rare. To prevent misdiagnosis as a primary salivary gland tumor, the use of combined immunohistochemistry is recommended for confirming the diagnosis. The prognosis for metastatic RCC is generally unfavorable. In this case, a combination of CN, metastasectomy, targeted therapy, and immunotherapy may be beneficial, but personalized treatment is essential. The adverse reactions experienced by the patient during treatment required the discontinuation of therapy due to their severity. Consequently, there is still a need for improved therapeutic approaches to alleviate patient distress and extend survival. This case provides a detailed description of the diagnostic criteria and treatment options (surgery, targeted therapy, and immunotherapy combined) for clear cell renal cell carcinoma presenting with tongue metastasis, along with the complete follow-up after treatment.

## Data Availability

The original contributions presented in the study are included in the article/supplementary material. Further inquiries can be directed to the corresponding author.
